# First person – Eli Matsell

**DOI:** 10.1242/dmm.050781

**Published:** 2024-04-24

## Abstract

First Person is a series of interviews with the first authors of a selection of papers published in Disease Models & Mechanisms, helping researchers promote themselves alongside their papers. Eli Matsell is first author on ‘ Functional and *in silico* analysis of ATP8A2 and other P4-ATPase variants associated with human genetic diseases’, published in DMM. Eli is a PhD student in the lab of Dr Robert Molday at the University of British Columbia, Vancouver, Canada, investigating the role that the P4-ATPase ATP8A2 plays in severe neurological disease.


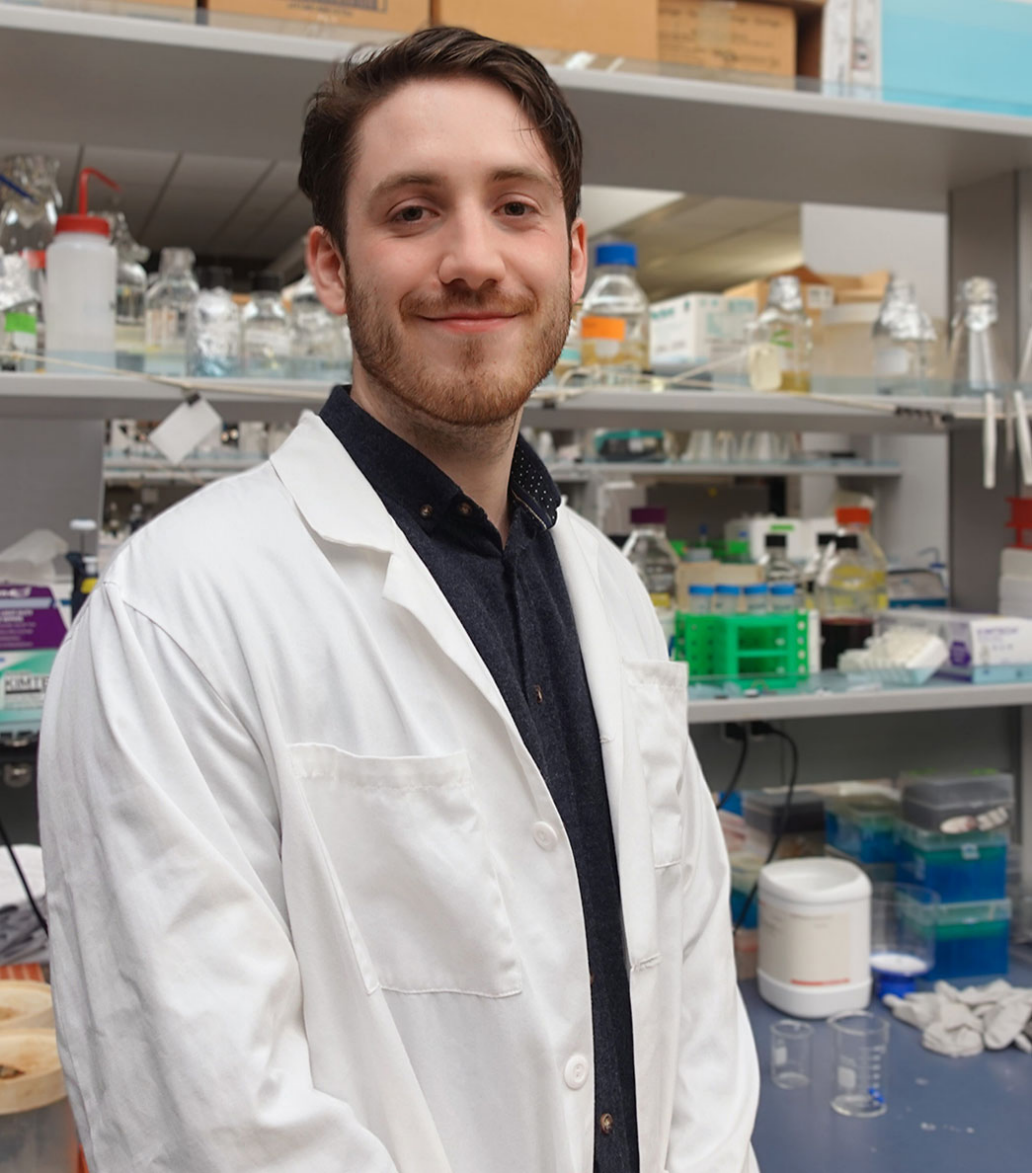


**Eli Matsell**

**How would you explain the main findings of your paper to non-scientific family and friends?**

*ATP8A2* is a gene that is associated with severe hereditary disease. This has several manifestations including, but not limited to, severe impairment of motor function, little to no ability to speak and delayed brain development. Although patient symptoms have been documented, the underlying mechanism by which ATP8A2 variants lead to disease in many cases is not well characterized and, as such, there are no treatments currently available. In this study, we characterized four distinct mutations in ATP8A2. Two of these mutations were found to be disease causing as they decreased the overall stability of the protein and decreasing the amount that is expressed within cells. Of the two other mutations characterized, one had a mild effect, potentially leading to milder symptoms, whereas the other had no detectable impact on the protein.

The tools used for this analysis were largely based on cellular tests in the wet lab; however, recent advances have developed predictive computer programs that have been used to guess whether a mutation in a gene will be disease causing. We decided to test some of these programs against our cellular tests in conjunction with other groups and we found a high degree of correlation between the predictive programs and our actual cell-based results. This indicates that these programs may be an effective method for detecting disease-causing mutations in proteins.

**What are the potential implications of these results for your field of research?**

The direct impact of this study is that we have gained information on how ATP8A2 mutations can lead to disease. To be able to solve a problem, it is important to understand what underlying mechanisms are being disrupted when the genetic variances arise. This research will hopefully someday help patients that are suffering from this disease to seek treatment designed to restore the proper function of ATP8A2.

Furthermore, identifying a high degree of correlation between our cell-based tests with those of the predictive programs has important implications. In the current time, computing power is only expanding and, as a result, predictive programs should hopefully become more and more accurate. If these programs are trained towards disease characterization, our diagnostic tools for determining disease-causing mutations and risks would expand greatly.

**What are the main advantages and drawbacks of the experimental system you have used as it relates to the disease you are investigating?**

The two experimental systems used in this study include both *in silico* and *in vivo* models. The *in vivo* system used involves expressing both wild-type and mutant constructs exogenously in given mammalian cell lines. The main advantage of this system is using a well-established model that has been shown to reliably model countless diseases. With any system, there are certain drawbacks, such as not necessarily being the native cell type for the given gene.

The *in silico* model system included programs that utilize algorithms to determine the effect of mutations on the stability of a program. The advantage of this system is that it is low cost, time efficient and very accessible. There are a few drawbacks when it comes to the *in silico* system, however. For example, the various programs used would contradict at certain points. Therefore, a biochemical or other testing method becomes essential for validation.

**Figure:**
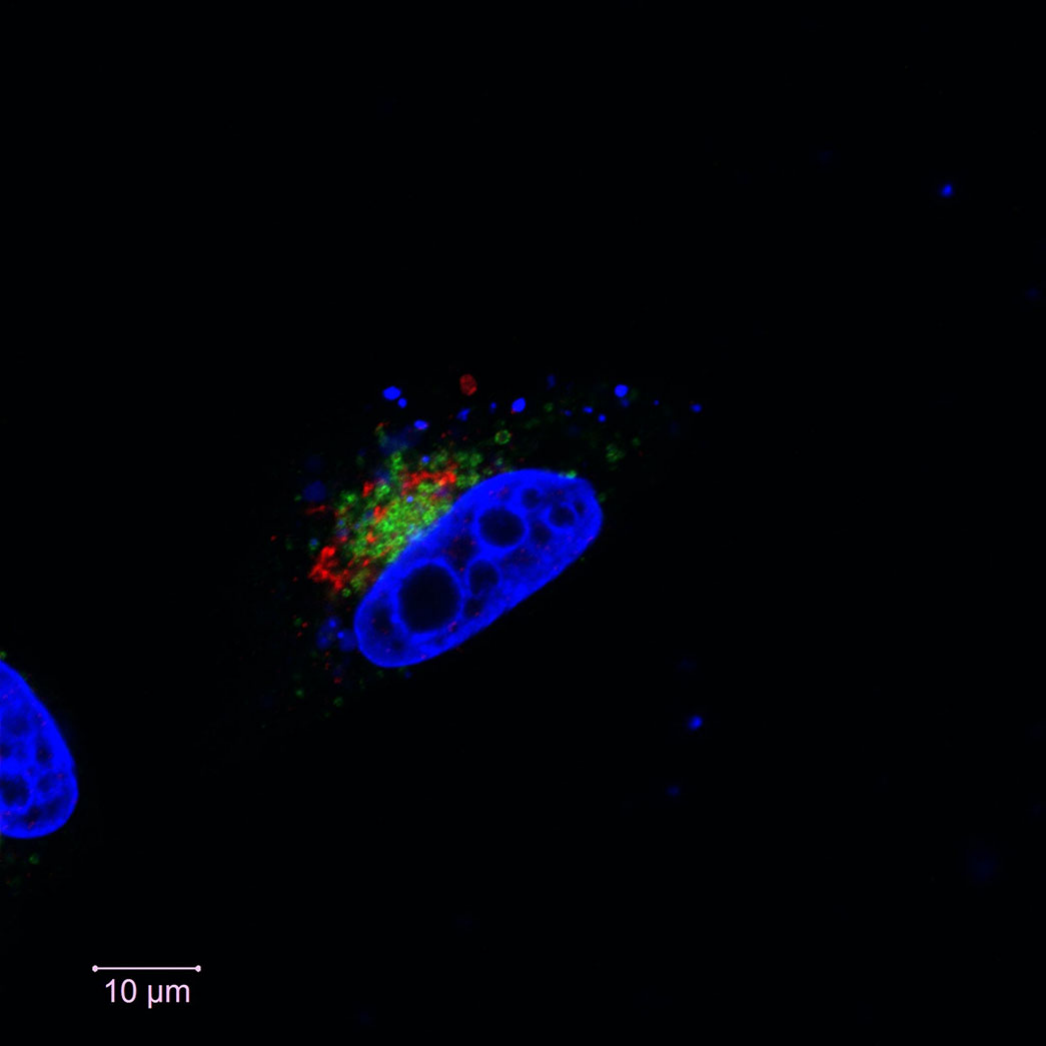
Immunofluorescence staining of HEK293T cells, showing the localization pattern of ATP8A2 (green) relative to the Golgi (red) and nucleus (blue) of the cell.

**What has surprised you the most while conducting your research?**

The most surprising finding for me during this study was how consistent the stability prediction programs were when compared to the protein expression levels measured for each mutant construct. When these comparisons were expanded to other P4-ATPases, the programs, specifically FoldX5, continued to have a high degree of consistency. This is interesting to me because if we can predict the stability changes before performing any wet lab work, this can save a lot of time and resources to get an effective diagnostic. For now, it appears that these predictive models still require biochemical and cell-based validation; however, the future is very exciting!

**What do you think is the most significant challenge impacting your research at this time and how will this be addressed over the next 10 years?**

The most significant challenge at this point in my opinion is that we don't fully understand the pathway that ATP8A2 aberrations are affecting. We know that patients with ATP8A2 variants suffer from severe neurological phenotypes and we know the biochemical function of the protein; however, what remain elusive are the downstream pathways that these mutations are affecting. Once these questions are revealed, a treatment can be designed specifically to restore the healthy function of the patient.

**What changes do you think could improve the professional lives of scientists?**

The university I study at goes to great lengths to offer mentorship opportunities for students, which I find extremely helpful. What I think could be offered to help the professional lives of early-stage scientists is more opportunities for mentorship in fields tangential to academia. I think this diversity of experience could help create a fuller picture for scientists that are beginning to plan the directions of their careers.

**What's next for you?**

In terms of research, I am interested in trying to further define the pathway that ATP8A2 disrupts when it is not functioning properly and trying to understand why it causes such severe disease. I'm hopeful that at some point, this will lead to the basis of treatment for patients with this disease. I am also hoping to write and defend my PhD thesis this upcoming year.
